# Expression of AIM2 is correlated with increased inflammation in chronic hepatitis B patients

**DOI:** 10.1186/s12985-015-0360-y

**Published:** 2015-08-20

**Authors:** Yongtao Han, Ziping Chen, Ruiping Hou, Daojie Yan, Changhong Liu, Shijun Chen, Xiaobo Li, Wenjun Du

**Affiliations:** Deparment of Pharmacy, Qilu Hospital, Shandong University, Jinan, China; Digestive Department, Shandong Provincial Qianfoshan Hospital, Shandong University, Jinan, China; Department of Infectious Disease, Hospital of Laiwu Affiliated to Taishan Medical College, Laiwu, China; Department of Liver Disease, Jinan Infectious Disease Hospital, Shandong University, Jinan, China; Center of Translational Medicine, Harbin Medical University, Harbin, Heilongjiang Province 150086 China; Digestive Department, Shandong Provincial Qianfoshan Hospital, School of Medicine, Shandong University, Jingshi Road 16766#, Jinan, 250014 China

## Abstract

**Background:**

The absent in melanoma 2 (AIM2), a cytosolic dsDNA inflammasome, can be activated by viral DNA to trigger caspase-1. Its role in immunopathology of chronic hepatitis B and C virus (HBV, HCV) infection is still largely unclear. In this study, the expression AIM2, and its downstream cytokines, caspase-1, IL-18 and IL-1β, in liver tissue of patients with chronic hepatitis B and C (CHB, CHC) were investigated.

**Methods:**

A total of 70 patients diagnosed with chronic hepatitis were enrolled, including 47 patients with CHB and 23 patients with CHC. A liver biopsy was taken from each patient, and immunohistochemistry was used to detect the expression of AIM2 and inflammatory factors caspase-1, IL-18, and IL-1β in the biopsy specimens. The relationship between AIM2 expression and these inflammatory factors was analyzed.

**Results:**

The expression of AIM2 in CHB patients (89.4 %) was significantly higher than in CHC patients (8.7 %), and among the CHB patients, the expression of AIM2 was significantly higher in the high HBV replication group (HBV DNA ≥ 1 × 10^5^copies/mL) than in the low HBV replication group (HBV DNA < 1 × 10^5^copies/mL). The expression of AIM2 was also correlated with HBV-associated inflammatory activity in CHB patients statistically. Additionally, AIM2 levels were positively correlated with the expression of caspase-1, IL-1β and IL-18 in CHB patients, which implied that the AIM2 expression is directly correlated with the inflammatory activity associated with CHB.

**Conclusions:**

AIM2 upregulation may be a component of HBV immunopathology. The underlying mechanism and possible signal pathway warrant further study.

## Introduction

Chronic hepatitis B virus (HBV) infection is a major clinical problem worldwide. The adverse outcomes caused by chronic HBV infection include cirrhosis, hepatic decomposition and hepatocellular carcinoma (HCC) [1]. In addition to the assumption that chronic liver injury is caused by the inflammatory processes initiated by host immunity when eliminating the virus, the pathological mechanisms underlying the progression of chronic hepatitis B(CHB) are not well understood. Current research is focused on the relationship between the adaptive immune response and the CHB pathological mechanism. However, there are relatively few studies that have targeted the relationship between innate immunity and CHB.

The innate immune system is the first line of defense against invading microbes and is activated by the engagement of germline-encoded pattern-recognition receptors (PRRs) [2]. PRRs recognize specific components of microbes and initiate the inflammatory pathway to prevent microbial invasion [2]. A major aspect of the inflammatory pathway is the activation of the inflammasome. The inflammasome is a multi-protein complex that can activate caspase-1, resulting in the cleavage and maturation of the pro-inflammatory cytokines interleukin-1β (IL-1β) and IL-18 [2]. Absent in melanoma 2 (AIM2) is one of the key genes orchestrating the formation of the inflammasome.

AIM2 is a member of the IFI20X-IFI16 protein family [3] and can bind to double-stranded DNA and to the adaptor molecule ASC (apoptosis-associated speck-like protein), which contains a caspase activation and recruitment domain. This complex then activates caspase-1 and leads to the formation of mature IL-1β and IL-18 [4]. Although AIM2 is known to be involved in the host defense against microbial invasion, its role in regulating the immune response to viruses, such as HBV, has not been well understood. A few studies have investigated caspase-1, IL-1β and IL-18 expression in patients with CHB, CHC, and other viral hepatitis [5–8]. HBV is a double-stranded DNA virus (HBV DNA) which has undergone replication in the nucleus of liver cells and re-entered the cytoplasm to finish packaging [9]. However, the expression of AIM2 and its relationship with liver inflammation in liver tissue in patients with CHB and CHC is still unclear.

In this study, we investigated the expression of AIM2 caspase-1, IL-1β, and IL-18 in liver tissue in patients with CHB or CHC.

## Patients and methods

### Patients

Subjects who fulfilled the following criteria were recruited into the groups: (1) age between 18 and 60 years old; (2) CHB or CHC without HAV, HDV, HEV or HIV co-infection; (3) without a history or current evidence of cancer; (4) alpha-fetoprotein (AFP) < 13.6 ng/mL; (5) without nucleotide/nucleosides or interferon treatment history; (6) without having used any heap-protective drugs or immune agents in the past 3 months; (7) without evidence of autoimmune diseases; and (8) agreement to liver biopsy. 47 patients with CHB and 23 patients with CHC were recruited between September 2009 and July 2011 at Jinan Infectious Disease Hospital and Qianfoshan Hospital of Shandong University, Shandong. All subjects received liver puncture biopsy under ultrasound guidance to attain hepatic tissue of no less than 2 cm in length for diagnosis and subsequent research. The study was approved by the ethics committee of Jinan Infectious Disease Hospital, and written informed consent for participation was obtained from each participator.

### Diagnosis of CHB and CHC

The diagnostic criteria for CHB were in accordance with the Asian-Pacific Consensus Statement on the Management of Chronic Hepatitis B [1, 10, 11].

The criteria for diagnosis of CHC followed the 2014 World Health Organization Guidelines for the screening, care and treatment of persons with hepatitis C infection [6].

### Immunohistochemistry and scoring

All liver tissue specimens were first fixed in 10 % formalin, and then the tissue was cut, dehydrated, dipped in wax, embedded and sectioned. These sections were then placed on slides, baked, placed into xylene, cleared of the wax, rehydrated using graded ethanol and immersed in 0.3 % hydrogen peroxide for 5 min to reduce non-specific background staining caused by endogenous peroxidase. The slides were then washed with PBS buffer three times for 5 min each, placed in citrate buffer solution at a pH of 6.0 and then into a high temperature pressure pot to recover the tissue antigen. After being heated, the slides were cooled and restored at room temperature, washed three more times in PBS buffer and incubated with AIM2, IL-18 and IL-1β primary antibodies (rabbit anti-human monoclonal antibody, Abcam, England) and caspase-1primary antibody (mouse anti-human monoclonal antibody, Santa Cruz Biotechnology Inc., USA). The slides were then placed in a 4 °C refrigerator overnight. The next day, the slides were washed with PBS buffer three times, each time lasting longer than 5 min, then incubated with the secondary antibody PV-9000 (universal antibody) at 37 °C for 10 min, washed with PBS buffer, and DAB staining was applied. The stain was terminated using running water, then the slides were washed with hydrochloric acid alcohol for differentiation. Lastly, the slides were washed with distilled water, cleared with xylene and mounted.

Appearance of a tan stain in the cytoplasm signaled positive expression of the protein. Scores were assigned based on percentage of positive cells and intensity of staining. Negative (−) was defined as no cells stained; mildly positive (+): 1–30 % of cells stained; moderately positive (++): 30–60 % of cells stained; and strongly positive (+++): more than 60 % of cells stained. Scores for stain intensity and percent of positivity were then added together, and a negative (−) was assigned for scores 0, mildly positive (+) for scores between 1 and 3, moderately positive (++) for scores between 4 and 6, and strongly positive (+++) for scores greater than 7.

### Histological assessment and quantification of fibrosis

Hepatic biopsies were obtained using 16 gauge disposable needles (D-78187, Gesingen, Germany). Liver specimens (median 22 mm, interquartile range [IQR] 20–23 mm) were stained with hematoxylin and eosin. Fibrosis staging (S) and inflammatory activity (G) were scored according to Precautionary and Curative Measures of Hepatitis Virus (PCMHV) [7]. For means of classification, the level of fibrosis in tissue specimens was divided into five stages, S0–S4: S0, no fibrosis; S1, portal fibrosis without septa but with minimal fibrosis of hepatic lobules; S2, periportal fibrosis with few septa and moderate fibrosis of hepatic lobules; S3, septal fibrosis with many septa and structural distortion of hepatic lobules; and S4, cirrhosis. Inflammatory activity was similarly divided into five grades, G0–G4: G0, no histologic necroinflammation; G1, portal inflammatory activity; G2, minimal patch necrosis; G3, moderate patch necrosis; and G4, severe patch necrosis. In accordance with the PCMVH, we defined significant fibrosis as having a score of two or greater (S2, 3, 4) and severe liver fibrosis as having a score of three or greater (S3, 4).

### Statistical analysis

The SPSS program (version 17.0) was used for analysis. Measurement data was described as mean ± standard deviation. Background factors were compared using the Student’s *t*-test (numerical data) or the Chi-square test (categorical data). Spearman’s two-tailed test was used for correlation analysis, and differences were regarded as significant if the *p* value was less than 0.05 on either side.

## Results

### Expression of AIM2 was significantly increased and highly correlated with HBV load in CHB patients

**Fig. 1:**
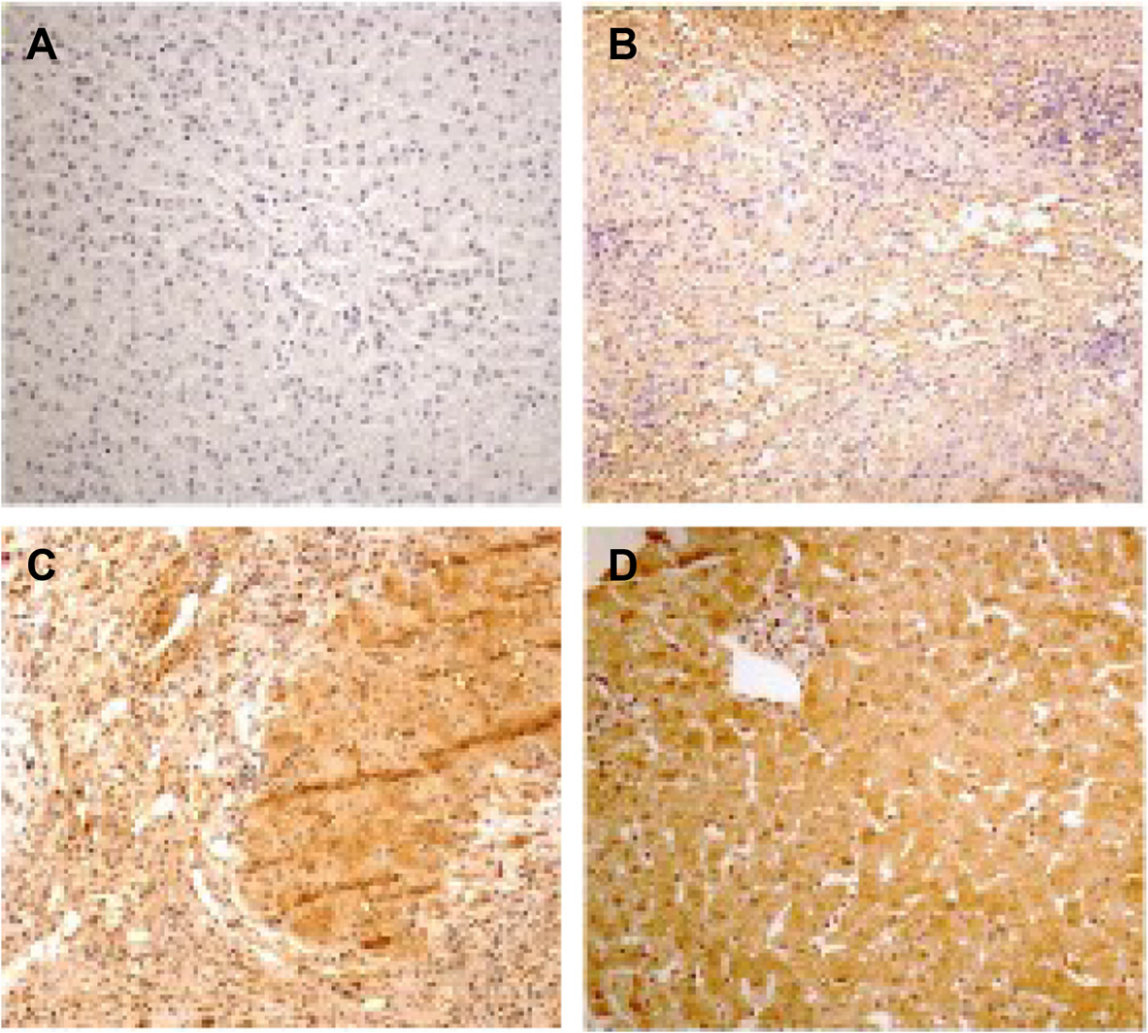
Immunostaining of AIM2 in the hepatocyte cytoplasm of CHB. **a** Negative expression of AIM2. **b** Weak positive of AIM2. **c** Moderate positive of AIM2. **d** Strong positive of AIM2. (Magnification of 400×)

We compared the expression of AIM2 in hepatic biopsies from 47 CHB patients and 23 CHC patients to determine whether AIM2 expression was correlated with HBV infection. Figure 1 shows the expression status of AIM2 ranging from negative (−) to strong positive (+++), as detected with immunohistochemistry, and suggests that AIM2 is exclusively expressed in the hepatic cellular cytoplasm, which is consistent with previous research [4]. To assess whether the observed results were indeed comparable, we also analyzed other clinical characteristics of both groups of patients (Table 1). No significant differences were observed between CHB patients and CHC patients in regard to their age (*p* = 0.075), gender (*p* = 0.81), liver function (ALT, *p* = 0.292; AST, *p* = 0.241; TB, *p* = 0.24; albumin, *p* = 0.165) or routine blood test (WBC, *p* = 0.381; PLT, *p* = 0.366). Statistical analysis revealed that the positive expression rate of AIM2 in CHB patients was significantly higher than that in CHC patients (89.4 vs. 8.7 %, *p* < 0.00). To further clarify the factors affecting the expression of AIM2 in CHB patients, we checked the potential influence of age, gender, liver function, and HBV DNA level on the expression of AIM2.

**Table 1:** Clinical characteristics and expression of AIM2 between CHB group and CHC group

	CHB	CHC	*P* value
	*n* = 47	*n* = 23	
Age (y)	38.2 ± 9.0	37.8 ± 9.6	0.750
Male, *n* (%)	30(63.8)	14(60.9)	0.810
ALT (IU/L)	64.1 ± 40.0	55.5 ± 31.9	0.292
AST (IU/L)	63.8 ± 25.0	58.4 ± 37.7	0.241
TB (μmol/mL)	18.6 ± 7.7	26.7 ± 9.7	0.240
ALB (g/L)	38.6 ± 11.9	39.8 ± 6.2	0.165
PTA (%)	85.7 ± 10.9	86.3 ± 12.4	0.722
WBC (10^9^/L)	6.4 ± 1.8	6.6 ± 2.0	0.381
PLT (10^9^/L)	137.7 ± 51.7	176.3 ± 55.6	0.366
AIM2, *n* (%)	42(89.4)	2 (8.7)	0.000

*AIM2* absent in melanoma, *CHB* chronic hepatitis B, *CHC* chronic hepatitis C, *ALT* alanine transaminase, *AST* aspartate aminotransferase, *TB* total Bilirubin, *ALB* albumin, *PTA* prothrombin activity, *WBC* white blood cell, *PLT* platelet

As summarized in Table 2, the results showed that AIM2 expression was not affected by age (*p* = 0.178) or gender (*p* = 0.148). The results also showed that AIM2 expression was significantly correlated with the level of ALT (*p* = 0.03) but not AST (*p* = 0.378). As for HBV status, we found that the expression of AIM2 was significantly higher in patients with high viral load (HBV-DNA ≥ 1 × 10^5^copies/mL) than in patients with low viral load (HBV-DNA < 1 × 10^5^ copies/mL) (*p* < 0.01); However, the expression of AIM2 was not related to hepatitis B e antigen (e-Ag) status (*p* = 0.825). These results suggest that the expression of AIM2 may be dependent on HBV load and may play certain roles in CHB.

**Table 2:** Correlation between AIM2, clinical characteristics, and lab findings in CHB group

		AIM2					
	*n*	−	+	++	+++	*x* ^2^	*p*
Male	30	1(3.3 %)	8(26.7 %)	10(33.3 %)	11(36.7 %)	5.351	0.148
Age(years)						4.918	0.178
20–40	30	3(10.0 %)	9(30.0 %)	7(23.3 %)	11(36.7 %)		
40–60	17	2(11.8 %)	1(5.9 %)	8(47.0 %)	6(35.3 %)		
ALT						39.987	0.030
ULN-2ULN	32	5(15.6 %)	7(21.9 %)	9(28.1 %)	11(34.4 %)		
2ULN-5ULN	15	0(0 %)	3(20.0 %)	6(40.0 %)	6(40.0 %)		
AST						36.487	0.378
ULN-2ULN	38	4(10.5 %)	9(23.7 %)	12(31.6 %)	13(34.2 %)		
2ULN-5ULN	9	1(11.1 %)	1(11.1 %)	3(33.3 %)	4(44.4 %)		
eAg						0.049	0.825
Positive	26	3(11.5 %)	5(19.2 %)	9(34.6 %)	9(34.6 %)		
Negative	21	2(9.5 %)	5(23.8 %)	6(28.6 %)	8(38.1 %)		
HBV DNA						25.96	<0.001
≥ 10^5^copies/mL	25	0(0 %)	0(0 %)	10(40.0 %)	15(60.0 %)		
< 10^5^copies/mL	22	5(20.0 %)	10(40.0 %)	5(20.0 %)	2(8.0 %)		

*CHB* chronic hepatitis B, *ALT* Alanine aminotransferase, *AST* Aspartate aminotransferase, *ULN* upper limits of normal

### Expression of AIM2 was correlated with inflammatory activity in CHB

Inflammatory activity and fibrosis staging are important pathological characteristics. High inflammatory activity indicates the severity of hepatic damage, and fibrosis staging is an indicator of the progression from a state of inflammation to cirrhosis. To reveal what role AIM2 plays in CHB patient liver damage, we analyzed the correlation between AIM2 expression and fibrosis and inflammation. The results showed that the expression of AIM2 was highly correlated with the degree of inflammation (*p* = 0.007) but not with the degree of fibrosis (*p* = 0.101) (Table 3).

**Table 3:** Correlation between expression of AIM2 and inflammatory activity and fibrosis staging in CHB

AIM2	G					S				
	0	1	2	3	4	0	1	2	3	4
	*n* = 0	*n* = 9	*n* = 14	*n* = 13	*n* = 11	*n* = 0	*n* = 13	*n* = 6	*n* = 8	*n* = 20
−	0	1	3	1	0	0	1	2	0	2
+	0	6	1	0	3	0	6	0	0	4
++	0	2	3	5	5	0	4	1	3	7
+++	0	0	7	7	3	0	2	3	5	7
*X*2	22.707					14.636				
*P*	0.007					0.101				

*G* Inflammatory activity, *S* Fibrosis staging

**Fig. 2:**
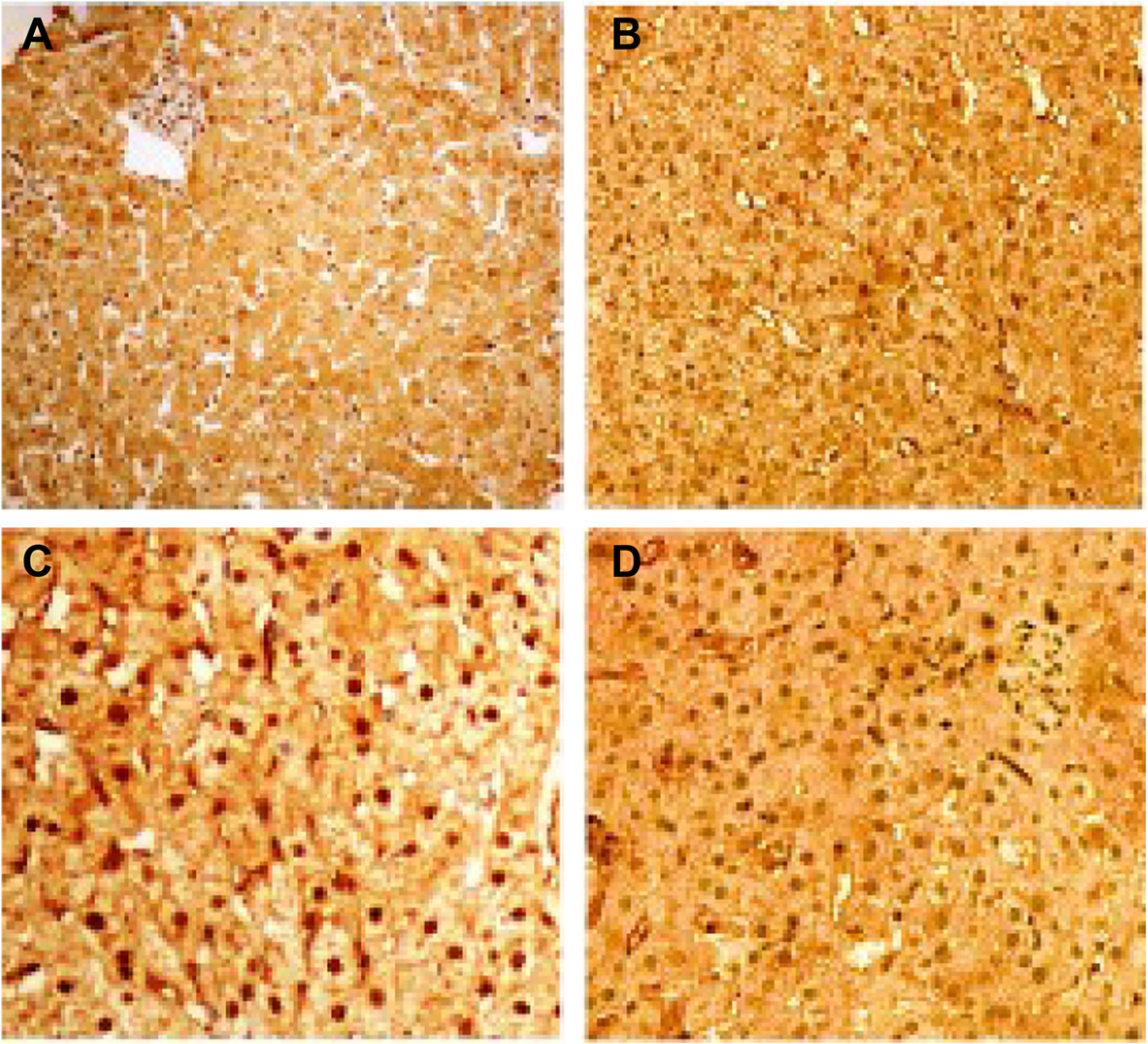
The strong expression of AIM2, caspase-1, IL-1β, and IL-18 in liver tissue of CHB. **a** Strong expression of AIM2. **b** Strong expression of caspase-1. **c** Strong expression of IL-1β. **d** Strong expression of IL-18. The four sections were taken from the same CHB patient (Magnification of 400×)

We further analyzed the correlation between AIM2 and caspase-1, IL-1β and IL-18 expression in CHB patients (Fig. 2). Statistical analysis revealed that the level of AIM2 was positively correlated with the level of caspase-1 (r_s_ = 0.738, *p* < 0.01), IL-1β (r_s_ = 0.527, *p* < 0.01) and IL-18 (r_s_ = 0.642, *p* < 0.01) (Table 4). Both IL-1β and IL-18, important factors contributing to the development of chronic hepatocellular inflammation. Figure 2 illustrates the expression of AIM2, caspase-1, IL-1β and IL-18 in these patients. These results suggested that the expression of AIM2 was specifically correlated with inflammation in CHB and that elevation of AIM2 corresponding to HBV infection or replication may contribute to the inflammatory damage associated with the development of CHB.

**Table 4:** Correlation between expression of AIM2 and Caspase-1, IL-1β and IL-18 in CHB group

AIM2	CHB											
	Capase-1				IL-1β				IL-18			
	−	+	++	+++	−	+	++	+++	−	+	++	+++
−	5	0	0	0	4	1	0	0	5	0	0	0
+	6	4	0	0	5	3	0	1	3	6	0	1
++	0	4	9	2	3	5	6	1	1	7	6	1
+++	1	0	2	14	1	5	7	4	1	3	7	6
r_s_	0.738				0.527				0.642			
*p*	< 0.01				< 0.01				< 0.01			

*AIM2* absent in melanom2, *CHB* chronic hepatitis B, *IL*-*1β* interleukin-1β, *IL*-*18* interleukin-18

## Discussion

AIM2 was first reported in malignant melanoma cell lines and was thought to act as a tumor suppressor by repressing NF-κB transcriptional activity [2]. Recent research has demonstrated that AIM2 plays an important role in the innate immune response through sensing potentially dangerous cytoplasmic DNA and inducing the formation of the ASC inflammasome, which then induces activation of caspase-1 and release of mature IL-1β and IL-18 [4]. As a receptor for cytoplasmic DNA, AIM2 can be activated by poly (dA:dT), plasmidic DNA and DNA from the bacterium *L. monocytogenes*, and even synthetic dsDNA [12]. Notably, dsDNA vaccinia virus has been shown to activate AIM2 [13].

Previous research indicated that AIM2 is highly expressed in the small intestine, spleen, peripheral white blood cells and testis [14]. In this study, we found that the expression of AIM2 in the liver is inducible, and AIM2 is exclusively expressed in hepatic cytoplasm. Furthermore, we found that the positive expression rate of AIM2 in CHB group was significantly higher than that in CHC control group. As neither clinical characteristics nor lab findings were statistically different between the two groups, this suggested that there is indeed a relationship between chronic HBV infection and AIM2 elevation.

In the CHB group, expression of AIM2 did not differ between e-Ag positive and e-Ag negative patients. Moreover, our results indicated that the expression of AIM2 in the high replication group (HBV DNA ≥ 1 × 10^5^copies/mL) was significantly higher than that in the low replication group (HBV DNA < 1 × 10^5^copies/mL), suggesting that high HBV load led to the increase in AIM2 expression. The relationship between high HBV load and progression of CHB infection has been tested showing an increasing incidence of HCC with high viral load [15–19]. While the function of AIM2 in HCC tumorigenesis is unclear, AIM2 has widely been believed to act as a tumor suppressor in a variety of cancer types via repressing NF-κB transcriptional activity [20–24]. However, recent research suggests that AIM2 may also act as an oncogene in some cancers [25]. Our results suggest that AIM2 expression may be related to the occurrence and development of HCC, a noteworthy finding to which further study should be devoted.

Our results showed various levels of AIM2 expression and inflammatory activity in patients with CHB. High levels of AIM2 were present in patient tissue specimens with a severe inflammatory grade, and low levels of AIM2 were found in mild cases, which suggest a relationship between the expression of AIM2 and liver inflammatory injury. Liver fibrosis is the accumulation of chronic inflammation in the liver. The observation that AIM2 is differentially expressed in regards to liver inflammatory grade versus stage of fibrosis suggests that AIM2 is closely associated with inflammation, not fibrotic development. Expression of AIM2 in groups with various levels of ALT and inflammatory activity was shown to be statistically different. ALT was regarded as the most important liver function index, reflecting hepatic inflammation for characteristics primarily in the hepatic cell; however, the degree of accuracy in appraising liver inflammatory activity still plays a small role in histodiagnosis. Because ALT values are easily affected by circumstance and condition, mildly elevated ALT might not truly translate to mild hepatic tissue damage [26]. Hepatic tissue tests are still regarded as the standard in appraising hepatic inflammatory damage. The relationship between the expression of AIM2 and hepatic tissue inflammatory grade is in fact stronger using these methods.

Our results showed that the expression of AIM2 is positively correlated with IL-1β and IL-18 expression in CHB patients and not correlated in the control fatty liver disease patient group. This might suggest that the mechanism of releasing IL-1β and IL-18 during CHB and CHC is different, although chronic inflammation was observed in both groups. The difference in correlation between AIM2 and caspse-1, IL-1β and IL-18 in CHB and CHC implies that the underlying cause for hepatic damage may be different between CHB and CHC. During the innate immune response to invading microbes, activated AIM2 may bind to asymptomatic carriers of hepatitis B virus (ASC), inducing activation of caspase-1 and releasing IL-1β and IL-18 [4]. Such a model of signal transfer was shown in our study. Within the CHB group, the expression level of AIM2 was positively correlated with the level of caspase-1, and the level of caspase-1 was positively correlated with levels of IL-1βand IL-18, further suggesting that this inflammation signal transfer pathway is related to AIM2 levels in CHB infections. IL-1β and IL-18 are both important intracellular cytokines belonging to the IL-1 superfamily [23]. Each binds to receptors and activates the downstream NF-ĸB signaling pathway, releasing inflammatory factors. Indeed, IL-1β and IL-18 release is known to cause tissue damage [24]. Collectively, the binding of HBV DNA to AIM2 may lead to the activation of caspase-1, inducing release ofIL-1β and IL-18, which in turn may play an important role in the pathogenesis of CHB.

## Conclusions

The present study showed the expression of AIM2 is correlated with increased inflammation in patients with chronic hepatitis B. AIM2 upregulation may be a component of HBV immunopathology. The underlying mechanism and possible signal pathway warrant further study.
